# Diagnostic Performance of Extrahepatic Protein Induced by Vitamin K Absence in the Hepatocellular Carcinoma: A Systematic Review and Meta-Analysis

**DOI:** 10.3390/diagnostics13050816

**Published:** 2023-02-21

**Authors:** Mirela Georgiana Perne, Adela-Viviana Sitar-Tăut, Teodora Gabriela Alexescu, Lorena Ciumărnean, Mircea-Vasile Milaciu, Sorina-Cezara Coste, Calin-Vasile Vlad, Angela Cozma, Dan-Andrei Sitar-Tăut, Olga Hilda Orăşan, Alexandra Crăciun

**Affiliations:** 1 4th Medical Clinic, Internal Medicine Department, Faculty of Medicine, “Iuliu Hatieganu” University of Medicine and Pharmacy Victor Babes Street, 400347 Cluj-Napoca, Romania; 2 Department of Business Information Systems, Faculty of Economics and Business Administration, Babeş-Bolyai University, 58–60 Theodor Mihaly Street, 400591 Cluj-Napoca, Romania; 3 Discipline of Medical Biochemistry, 3rd Department—Molecular Sciences, Faculty of Medicine, “Iuliu Haţieganu” University of Medicine and Pharmacy, Victor Babes Street, 400347 Cluj-Napoca, Romania

**Keywords:** hepatocellular carcinoma, PIVKA II, alpha-fetoprotein, diagnosis

## Abstract

Background and Objectives: the early diagnosis of hepatocellular carcinoma (HCC) benefits from the use of alpha-fetoprotein (AFP) together with imaging diagnosis using abdominal ultrasonography, CT, and MRI, leading to improved early detection of HCC. A lot of progress has been made in the field, but some cases are missed or late diagnosed in advanced stages of the disease. Therefore, new tools (serum markers, imagistic technics) are continually being reconsidered. Serum alpha-fetoprotein (AFP), protein induced by vitamin K absence or antagonist II (PIVKA II) diagnostic accuracy for HCC (global and early disease) has been investigated (in a separate or cumulative way). The purpose of the present study was to determine the performance of PIVKA II compared to AFP. Materials and Methods: systematic research was conducted in PubMed, Web of Science, Embase, Medline and the Cochrane Central Register of Controlled Trials, taking into consideration articles published between 2018 and 2022. Results: a total number of 37 studies (5037 patients with HCC vs. 8199 patients—control group) have been included in the meta-analysis. PIVKA II presented a better diagnostic accuracy in HCC diagnostic vs. alpha-fetoprotein (global PIVKA II AUROC 0.851 vs. AFP AUROC 0.808, respectively, 0.790 vs. 0.740 in early HCC cases). The conclusion from a clinical point of view, concomitant use of PIVKA II and AFP can bring useful information, added to that brought by ultrasound examination.

## 1. Introduction

Hepatocellular carcinoma (HCC) is the most widespread histological subtype of primary liver cancer (accounting for approximately 90% of all cases [1]), with an increasing incidence [2]; at the same time, it is currently recognized as the third most common cause of death worldwide [3,4,5]. Unfortunately, at the time of diagnosis, a small percentage of patients are eligible for curative treatment, the most common cause being an advanced tumor stage [6].

Studies in the literature have shown that chronic viral hepatitis B and C, autoimmune hepatitis, nonalcoholic steatohepatitis, and genetic and epigenetic changes are the main risk factors for the development of hepatocellular carcinoma HCC [7,8,9,10,11]. The prognosis of patients with advanced liver disease or cirrhosis (regardless of etiology), even in those responding to antiviral treatment, is influenced by the appearance of HCC [4,5].

Hepatocarcinogenesis is a gradual process characterized by genetic and molecular changes in the hepatocytes, followed over time by the appearance of a neoplastic lesion detectable by imaging.

Over the last ten years, the focus has shifted toward early HCC detection, this being known to influence treatment, curability of the disease and long-term survival [1,12]. Today, treatment strategies have diversified, including surgical resection, drug treatment, percutaneous treatment (ablation or chemoembolization) and liver transplant. Current guidelines recommend that in at-risk patients, the screening strategy should be based on complementary examination [13] like serum alpha-fetoprotein determination and abdominal ultrasonography at 3–6 months for early detection of HCC in groups of patients at risk [11,14,15,16,17,18,19].

Abdominal ultrasonography, however, remains an investigation with important limitations (patient-dependent or operator-dependent), being unable to detect small tumor formations with undesirable accuracy [11,20,21].

One of the traditional serum tumor markers for detecting and tracking HCC commonly used is alpha-fetoprotein (AFP) [22,23,24], its role evolving over time [23]. However, despite being widely used—being a non-invasive and affordable method—according to studies in the literature, it has suboptimal performance for the early detection of hepatocarcinoma [25]. Typically, a serum AFP level of 20 ng/mL is considered a borderline value to differentiate HCC from non-tumoral pathology [13]. Therefore, AFP has a high rate of false-negative results—approximately 40%—in the detection of early-stage tumors [16,26,27,28,29,30]. At the same time, a high proportion of patients with liver cirrhosis or chronic viral hepatitis without associated HCC frequently show false-positive results [23]. In these conditions, the diagnostic accuracy of determining AFP serum levels is unsatisfactory, due to low sensitivity (estimated between 39% and 64%) and specificity (in the 76–91% range).

New classes of biomarkers with promising results in the early detection of HCC, such as microRNAs (miRNAs) [1,31,32], PIVKA II, known also as des-gamma-carboxy-prothrombin (DCP), or the fucosylated fraction of the AFP fraction (AFP-L3), stanniocalcin 2, APEX1 [33,34,35,36] have now been described. However, their use in medical practice may be limited by the absence of standardized analytical determining methods.

PIVKA II (first described in 1984 [37]) as an immature form of prothrombin (synthesized in the liver) can be used to estimate hepatic vitamin K status. PIVKA II seems to be a more suitable biomarker for the detection of vitamin K deficiency [38]. PIVKA II measurement shows increased sensitivity and specificity compared to other methods conventionally used (standard coagulation tests such as prothrombin time and activated partial thromboplastin time) to assess a deficiency of vitamin K [39].

In the absence of vitamin K, when its action is antagonized, or in the presence of neoplastic cells, PIVKA II is released into the blood.

In patients with gastrointestinal malignancies, PIVKA II levels were increased in most patients, with previous data pointing out a good sensitivity, respectively, the specificity for PIVKA II in gastrointestinal neoplastic disorders diagnostic: 78.67% and 90.67% in pancreatic adenocarcinoma, 83.93% and 91.50% in HCC. For establishing the association of serum levels of PIVKA II with colorectal cancer, additional studies are needed [40]. Just one case report referring to the colorectal neoplasm with secondary dissemination to the liver and the presence of increased serum levels of PIVKA II was found [40]. At the same time, previously published studies showed that PIVKA II is an effective and specific biomarker for HCC. Some researchers have demonstrated that PIVKA II levels reflect the oncogenesis and progression of HCC [41]. However, the efficacy of PIVKA II has not been sufficiently studied.

Serum and tissue overexpression of PIVKA II may be a specific tumor marker for HCC, showing promising results (no matter the hepatocarcinoma stage—62.5% sensitivity and 85.5% specificity), but also indicating a poor prognosis, such as the presence of microvascular invasion and intrahepatic metastases [39,42]. According to current studies, elevated serum level of PIVKA II are associated with tumor size, microvascular invasion, and possible recurrence of HCC [12,22,43,44,45]. What differentiates PIVKA II from AFP is that the value of the former is not affected by liver disease activity [12].

In view of the above, the difficulty of performing adequate screening for HCC (to detect early cases), new screening methods are being examined. Current studies aim at comparative and summative evaluation of different methods, with Japan and other countries [46,47] implementing simultaneous determination of PIVKA II and AFP as a screening method to monitor patients at high risk of developing hepatocellular carcinoma [48].

To date, results on the diagnostic performance of PIVKA II in comparison to or in combination with AFP are conflicting. The available data come mainly from studies involving Asian patients [46,49], with results from Western studies limited by a relatively small sample size. Published studies (with the exception of the most recently published ones) have been systematized into past published meta-analyses, evaluating the accuracy of HCC detection by serum determination of PIVKA II and AFP biomarkers, alone or in combination in patients at risk of tumor development [44,45,48,49].

In the present one, the most recently published studies for establishing the role of PIVKA-II versus AFP (globally, but also in a relationship with the HCC stage) were taken into consideration.

Knowledge of this topic is needed for better screening and diagnosis of at-risk HCC patients. The aim of this work was to extend the knowledge of comparative evaluation of PIVKA II and AFP HCC diagnostic values, especially in early HCC patients.

## 2. Materials and Methods

Search strategy: literature screening for meta-analysis. A systematic search was conducted for the interval from 1 January 2018 to 4 September 2022. Searches for relevant studies were mainly conducted in PubMed, Web of Science, Embase, Medline and the Cochrane Central Register of Controlled Trials.

All publications from the databases mentioned above were reviewed, using the terms (((‘descarboxyprothrombin’ OR des-gamma-carboxy prothrombin) AND (‘liver cell carcinoma’ OR ‘hepatocellular carcinoma’) AND ‘cancer diagnosis’) OR (‘pivka’ AND (‘liver cell carcinoma’ OR ‘hepatocellular carcinoma’) AND ‘cancer diagnosis’) OR (‘DCP’ AND (‘liver cell carcinoma’ OR ‘hepatocellular carcinoma’) AND ‘cancer diagnosis’)) AND ((‘alphafetoprotein’ OR afp OR ‘alpha fetoprotein’ OR alfa-fetoprotein) AND (‘liver cell carcinoma’ OR ‘hepatocellular carcinoma’) AND ‘cancer diagnosis’). Only human studies from the mentioned period were selected for screening.

Rigorous research of the papers was performed. Two main investigators performed independent literature research in order to identify the previously published papers. All useful papers were read by both investigators, even those with negative results.

Duplicates were removed. Only articles written in English that had abstracts were taken into consideration. Articles presented just as abstracts or conference presentations, reviews, systematic reviews, meta-analyses, editorials and in vitro studies were excluded. The quality assessment of diagnostic accuracy studies (QUADAS) was applied to evaluate the selected studies from a quality point of view.

The following data were extracted from the articles studied: title, authors, year of publication, study identification item, country, number of locations where the study was conducted, number of patients included (with HCC vs. without HCC, respectively, early HCC cases), study design, etiology of liver disease; for both PIVKA II and AFP, the AUROC (overall and for early HCC cases), sensitivity and specificity were followed.

**Figure 1:**
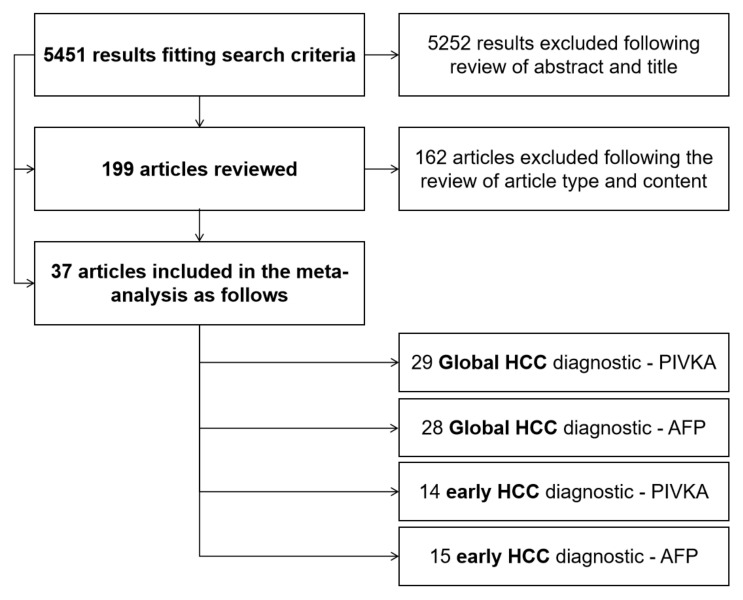
Flow diagram of the literature search strategy.

A flow diagram of the literature search strategy and study selection process is summarized in Figure 1.

According to the literature, at this moment, two tumor staging systems are used to define the extent of HCC—BCLC (Barcelona clinic liver cancer staging) staging [50,51], respectively, the 8th edition American Joint Committee on Cancer tumor–node–metastasis (TNM) staging [52]. BCLC stage 0 is defined as the tumor being less than 2 cm, performance status = 0 and the liver working normally (Child–Pugh A). BCLC stage A is defined in patients presenting single tumors of any size or 3 nodules < 3 cm in diameter, performance status = 0 and Child–Pugh class A or B.

In this meta-analysis, early-stage HCC was defined as BCLC stage 0/A and/or 8th edition TNM stage I (depending on the data reported by the included studies).

### Statistical Analysis

MedCalc software version 20.115 (Ostend, Belgium) was used for performing the meta-analysis. Using for every study each AUC value and the corresponding standard error (SE), the weighted summary AUC (sAUC) was calculated. Most of the studies did not report the standard error for AUROC. The formula used for SE (AUC) calculation was the one proposed by Hanley and McNeil (1982)—presented in Formula (1).

The publication bias was assessed using funnel plots. Forrest plots showing the overall effect were constructed. Taking into consideration the presence or absence of heterogeneity, a fixed or random effects model was preferred. An I2 value >25% was considered indicative of heterogeneity.

Formula (1)—AUROC standard error estimation

(1) $$SE\left(AUC\right)=\sqrt{\frac{AUC\left(1-AUC\right)+\left(N_{1}-1\right)\left(Q_{1}-AUC^{2}\right)+\left(N_{2}-1\right)\left(Q_{2}-AUC^{2}\right)}{N_{1}N_{2}}}$$

where $Q_{1}=\frac{AUC}{2-AUC}$; $Q_{2}=\frac{2AUC}{1+AUC}$; N_1_—positive group (with HCC); N_2_—negative group (without HCC).

A *p* value < 0.05 was considered statistically significant.

## 3. Results

A total number of 37 studies were included in the meta-analysis. Overall, 13,236 patients were included: 5037 patients with HCC (case group) vs. 8199 patients (the control group). The control group was represented by healthy patients (without previous liver diseases), chronic hepatitis B or C, liver cirrhosis or at-risk condition patients. Patients with HCC were divided depending on their HCC stage—1513 early HCC. Complete data about the included studies are presented in Table 1.

For each study included, the performances of PIVKA II and AFP were reported in Table 2 and Table 3 (global and in early HCC cases). Sensibility and specificity for PIVKA II and AFP were also reported.

**Figure 2:**
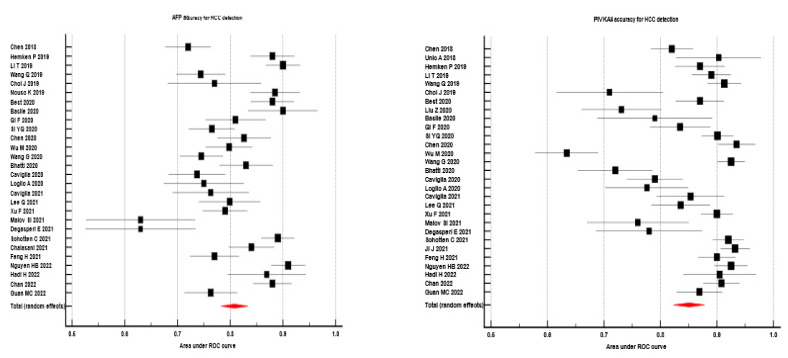
The capacity of AFP vs. PIVKA II to diagnose HCC—global–Forrest plot of AFP vs. PIVKA II accuracy for **HCC detection;** Left panel of figure-red marker–AUROC for AFP determined by meta-analysis; Right panel of figure-red marker–AUROC for PIVKA II determined by meta-analysis. References (Chen 2018 [66], Hemken P 2019 [61], Li T 2019 [78], Wang Q 2019 [77], Choi J 2019 [54], Nouso K 2019 [60], Best 2020 [55], Basile 2020 [12], Qi F 2020 [71], Si YQ 2020 [74], Chen 2020 [82], Wu M 2020 [56], Wang G 2020 [76], Bhatti 2020 [6], Caviglia 2020 [1], Loglio A 2020 [65], Caviglia 2021 [21], Lee Q 2021 [81], Xu F 2021 [83], Malov SI 2021 [57], Degasperi E 2021 [69], Schotten C 2021 [53], Chalasani 2021 [59], Feng H 2021 [79], Nguyen HB 2022 [80], Hadi H 2022 [85], Chan 2022 [58], Guan MC 2022 [73], Unic A 2018 [62], Li T 2019 [78]; Liu Z 2020 [63], Wang G 2020 [76], Ji J 2021 [75].

The sAUC of AFP, respectively PIVKA II for the discrimination between patients with HCC and those without, were 0.808 (95% CI 0.782 to 0.834) vs. 0.851 (95% CI 0.823 to 0.878)-data were reported in Figure 2. Considering that the studies showed heterogeneity (in both cases), random effects models were applied.

**Figure 3:**
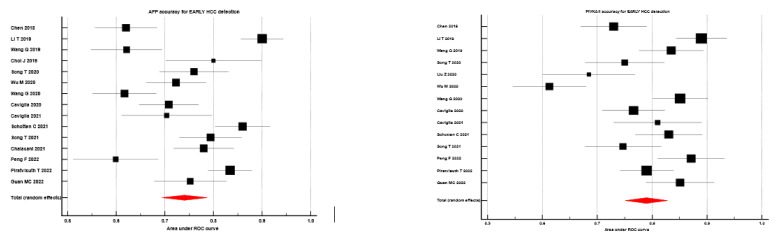
Forrest plot of AFP vs. PIVKA II accuracy for EARLY HCC detection; Left panel of figure-red marker–AUROC for AFP determined by meta-analysis; right panel of figure-red marker–AUROC for PIVKA II determined by meta-analysis. References Chen 2018 [66], Li T 2019 [78], Wang Q 2019 [77], Choi J 2019 [54], Song T 2021 [64], Wu M 2020 [56], Wang G 2020 [76], Caviglia 2020 [1], Caviglia 2021 [21], Schotten C 2021 [53], Song T 2020 [68], Chalasani 2021 [59], Peng F 2022 [84], Piratvisuth T 2022 [67], Guan MC 2022 [73], Liu Z 2020 [63], Schotten C 2021 [53], Song T 2020 [68].

Taking into consideration the capacity of discrimination in early HCC cases, the sAUC of AFP, respectively, PIVKA II were 0.740 (CI 95% 0.694 to 0.787), respectively, 0.790 (95% CI 0.751 to 0.828)–data were reported in Figure 3.

Some of the studies reported at the same time for AFP and PIVKA II; also, there were some studies reporting global for HCC, but also for early HCC; AFP = alpha-fetoprotein; PIVKA II = protein induced by vitamin K absence or antagonist-II.

## 4. Discussion

In our days, the neoplastic diseases show an increasing prevalence, with HCC being more and more frequently diagnosed, even in young patients. Lifestyle changes with an increased incidence of nonalcoholic steatohepatitis, chronic viral hepatitis and autoimmune hepatitis increase the risk of HCC, being responsible for HCC appearance.

A lot of progress has been made in HCC diagnosis, but some cases are missed or late diagnosed in advanced stages of the disease.

Therefore, new tools (serum markers, performant imagistic technics) are continually being reconsidered.

For decades, AFP has been widely used as a tumor marker in the surveillance of populations at high risk of developing HCC, but some limitations are well known. The reported sensitivity and specificity of this biomarker (40–65%, 76–96%, respectively) differ significantly depending on the characteristics of the studied group [22,86]. AFP serum values were often elevated in patients with chronic liver disease or cirrhosis without HCC [22].

All current guidelines recommend the additional use of imaging diagnosis in order to improve the diagnostic accuracy. Ultrasonography, CT or MRI present limitations, sometimes encountering difficulties in small lesion diagnosis. In view of these data, AFP and ultrasonography have been used together to improve diagnostic sensitivity in medical practice [3,86,87,88,89,90], but accuracy for the moment remains uncertain [91]. Under these conditions, HCC screening can be improved to detect neoplastic lesions at early stages. To date, several promising serum tumor markers with the potential for early diagnosis and surveillance of HCC have been proposed [3,21,44,45,86,87,88,89,90,92,93] of which PIVKA II appears to be the most promising, with recently published data on its performance (alone or in combination with AFP or ultrasonography).

No clear PIVKA II cut-offs for HCC, respectively, for early HCC diagnosis were already established. Supplementary, different methods are used for biomarker determination—so, for clarifying these aspects, more data need to be published. To this moment, to our best knowledge, clinical and laboratory factors influencing the PIVKA II values have not been exhaustively investigated. The current meta-analysis brings to attention new data about the usefulness and ability of PIVKA II to detect HCC. Literature is scarce in revealing the role of PIVKA II versus AFP. The paper provides an overview of recently published data about the role of PIVKA II vs. AFP in HCC diagnosis. In this meta-analysis, PIVKA II presented greater accuracy for HCC diagnosis, taking into consideration all cases (0.851 vs. 0.808), but also in early HCC cases (0.790 vs. 0.740). The reported results (the better discriminatory value of PIVKA II) are in line with those reported by Caviglia [3] (11 studies, published between 2011 and 2017), Chen H [94](27 studies, 2000–2016), Fan J [7] (40 studies, up to December 31, 2018), Fang Y [95] (28 studies, 2015–2021), Xing H [87] (31 studies, up to December 20, 2017). Also, the reported calculated AUROCs were approximately similar to those reported in previously mentioned studies. There also has been published some meta-analysis evaluating just one of the two biomarkers (PIVKA II or AFP), the results regarding the AUROC values being consistent with the results of this study [89,96,97].

A novel perspective brought to attention a parallel with the standard marker used (AFP)—higher accuracy for PIVKA II being found in the early diagnosis of HCC. Similar data have been published by Xing [87]. The results of this study highlight the possible role of PIVKA II in providing new data, useful for daily medical practice. A recently published study (just a few days ago, not included in the meta-analysis) also revealed that PIVKA II had a better predictive performance vs. AFP global and in early-HCC (the reported registered values being approximately similar to these ones [22]). The results of the study are supporting others’ recommendations that PIVKA II can have usefulness in early HCC diagnosis in the incipient moments [66].

It was impossible to determine the PIVKA II and AFP performances, depending on the etiology of the liver diseases, with mixed etiology being taken into consideration. Frequently, the studies do not make a difference according to the liver disease etiology; in most of the cases, the reported results are globally calculated.

The 0 and A HCC BCLC classes were taken into consideration in a unitary way, which must be mentioned. Of course, that is a discrepancy between the two classes regarding the following treatment, but for the moment it was not possible to perform a detailed, stratified analysis.

Due to the heterogeneity of the reported studies (determined by the diversity of study populations in different countries, methodology used and sample size), these findings might not be representative of all populations—further research is needed.

Stratified analysis depending on the gender, ethnicity, age or liver disease type and stage represents an area to be explored further. More data should be published regarding the cut-off values, for a unitary approach regarding HCC diagnosis.

This study provides the backbone for a future meta-analysis in order to evaluate the accuracy of PIVKA II and AFP association in HCC diagnosis. Of the listed studies, just a few of them reported combined accuracy. In addition, future studies on the topic are recommended to determine the serum values of PIVKA II after HCC treatment (surgical or chemotherapy), theoretically bringing useful information for monitoring treatment results, for predicting diagnosis, relapse and survival.

## 5. Conclusions

These results provide a significant step toward the diagnosis of HCC by determining the serum value of vitamin-K-dependent proteins used as tumor biomarkers, along with other paraclinical examinations.

From a clinical and practical point of view, the use of PIVKA II concomitantly or instead of AFP is bringing useful information, added to those reported by ultrasound examination. Probably the emerging role of PIVKA II is in patients with previous hepatic diseases (hepatitis, cirrhosis) where AFP limitations are well-known.

This study provides the backbone for future studies on the relationship with an earlier diagnosis of hepatocarcinoma.

## Figures and Tables

**Table 1:** All studies included in meta-analysis (for HCC discrimination or for early HCC discrimination).

Study	Period	Country	Study-Type	Patients	No	Cut-Off PIVKA II	Cut-Off AFP
Schotten C 2021 [53]	2008–2020	Germany	Retrospective study	182 patients with HBV, 223 with HCV, 168 with other etiology, HCC—52 HBV, 84 HCV and 60	573	NA	20 ng/mL
Choi J 2019 [54]	NA	Korea	Matched case-control	42 HCC; 168 cirrhosis or chronic B hepatitis	210	20 mAU/mL	5 ng/mL
Best 2020 [55]	2005–2016	Germany Japan	Multicenter case-control study	126 patients with HCC; 231 patients without HCC, NASH controls	357	NA	NA
Wu M 2020 [56]	NA	China	Observational study	176 healthy, CHB, LC; 198 very early HCC + early HCC + advance and HCC	374	NA	NA
Malov SI 2021 [57]	NA	Russia	Case-control study	110 patients with chronic hepatitis C in the stage of liver cirrhosis 55 without HCC; 55 with HCC	110	20 ng/mL	20 ng/mL
Chan 2022 [58]	NA	China Germany Japan Thailand.	Multicenter prospective study	168 HCC, 208 patients without HCC with an at-risk condition—cirrhosis, non-cirrhotic chronic hepatitis B virus (HBV), non-cirrhotic chronic hepatitis C virus (HCV), NASH	376	28.4 ng/mL	20 ng/mL
Chalasani 2021 [59]	NA	ClinicalTrials.gov	International, multicenter, case-control study	136 HCC, 404 controls at-risk patients with chronic liver disease—HCV, NAFLD, ASH, HBV, other chronic liver disease	540	NA	20 ng/mL
Basile 2020 [12]	NA	Italy	Case-control study	20 metabolic, 40 viral newly diagnosed HCC, 20 healthy subjects	80	38 mAU/mL,	3.5 ng/mL
Nouso K 2019 [60]	2001–2016	Japan	Case-control study	172 tumor-free diabetes mellitus, 93 consecutive NBNC-HCC patients	265	20 mAU/mL	3 ng/mL
Hemken P 2019 [61]	2003–2016	SUA	Retrospective case-control study	119 HCC, 215 nonmalignant liver disease, 34 healthy	368	NA	NA
Unic A 2018 [62]	2009–2011	Croatia	Consecutively recruited study	20 healthy volunteers, 31 patients with alcoholic liver cirrhosis, 32 patients with HCC.	83	108 mAU/mL	NA
Liu Z 2020 [63]	2010–2018	China	Retrospective study	87 AFP-negative HBV-related HCC, 123 control cases—benign liver disease, chronic HBV infection or liver cirrhosis	210	45 mAU/mL	NA
Song T 2021 [64]	2010–2020	China	Cross-sectional study	48 chronic HBV infection (CHB), 64 liver cirrhosis (LC), 33 early-stage CHB-HCC, 55 early-stage LC-HCC.	200	44 mAU/mL	5 ng/mL
Loglio A 2020 [65]	2010–2020	Italy	Cross-sectional, case-control study	64 with HCC (cases), 148 HCC-free (control)	212	48 mAU/mL	4.2 ng/mL
Caviglia 2020 [1]	2012–2018	Italy	Cross-sectional study	149 HCC, 200 cirrhosis of viral etiology	349	73 mAU/mL	9.7 ng/mL
Caviglia 2021 [21]	2012–2020	Italy	Retrospective case-control study	191 NAFLD patients cohort, 72 of whom had a diagnosis of HCC, 119 non-HCC patients	191	56 mAU/mL	4.4 ng/mL
Chen H 2018 [66]	2013–2014	China	Cross-sectional, consecutively recruited study	202 HCC patients, 226 liver cirrhosis patients, 215 chronic hepatitis B virus-infected 203 healthy	846	NA	NA
Piratvisuth T 2022 [67]	2014–2016	China Germany Spain Thailand	Case-control study	308 HCC, 740 chronic liver disease—cirrhotic liver disease independent of etiology, noncirrhotic NASH, chronic HBV infection, chronic HCV infection	1048	NA	NA
Song T 2020 [68]	2014–2017	China	Prospective study	100 HCC in patients with hepatitis B virus (HBV)—associated liver cirrhosis (LC), 67 LC	167	38 mAU/mL	10 ng/mL
Degasperi E 2021 [69]	2014–2019	Italy	Retrospective study	34 HCC, 366 non-HCC patients	400	47 mAU/mL	17 ng/mL
Wu J 2018 [70]	2016–2017	China	Case-control study	51 healthy, 37 chronic hepatitis, 43 cirrhotic; 143 HCC	274	40 mAU/mL	10 ng/mL
Qi F 2020 [71]	2016–2018	China	Prospective study	120 HCC, 89 chronic liver disease—nonviral, autoimmune, fatty-liver, HBV, HCV	209	33.08 mAU/mL	11.88 ng/mL
Li Y 2019 [72]	2016–2018	China	Retrospective study	Group 1 non-cancer, Group 2 primary cancer in liver patients—not available the numbers	1190	60.5 mAU/mL	NA
Guan MC 2022 [73]	2016–2020	China	Retrospective observational study	139 HCC, 345 NAFLD	484	40 mAU/mL	20 ng/mL
Si YQ 2020 [74]	2017–2018	China	Case-control study	266 cases with HBV-related HCC, 87 HBV DNA-positive benign liver disease, 80 healthy individuals	433	41.74 mAU/mL	21.8 ng/mL
Ji J 2021 [75]	2017–2018	China	Cross-sectional, multicenter study	183 HCC-CHB- and HBV-related, 312 cases were chronic hepatitis and 289 cases were cirrhosis	784	40 mAU/mL	20 ng/mL
Wang G 2020 [76]	2017–2018	China	Retrospective study	234 HBV-related HCC, 396 patients with chronic hepatitis B (CHB)	630	87.63 mAU/mL	499.80 ng/mL
Bhatti 2020 [6]	2017–2019	Pakistan	Retrospective study	Cirrhotic patients, surgical candidates—176 HCC, 68 non-HCC	244	250 mAU/mL	7.6 ng/mL
Wang Q 2019 [77]	2017–2019	China	Retrospective study	176 HBV-related HCC, 359 patients with chronic hepatitis B.	535	162.22 mAU/mL	145.65 ng/mL
Li T 2019 [78]	2017–2019	China	Case-control study.	169 newly diagnosed early HCC, 242 LC without HCC	411	NA	NA
Feng H 2021 [79]	2017–2019	China	Case-control study.	168 HCC patients,150 benign liver disease, 153 healthy controls	469	35.60 mAU/mL	17.76 ng/mL
Nguyen HB 2022 [80]	2018–2019	Vietnam	Case-control study.	170 chronic hepatitis B virus, hepatitis C virus, 170 HCC	340	29.01 mAU/mL	5.1 ng/mL
Lee Q 2021 [81]	2018–2020	China	Prospective study	158 primary HCC in chronic hepatitis B, 62—chronic hepatitis B	220	34.92 mAU/mL	9.10 ng/mL
Chen J 2020 [82]	2019	China	Case-control study	110 patients HBV-associated HCC, 70 HBV-related LC, 70 CBH, 110 healthy	360	51.00 mAU/mL	5.65 ng/mL
Xu F 2021 [83]	2019	China	Retrospective study	308 HCC, 60 HBV-related LC, 60 benign liver disease	428	40 mAU/mL	25 ng/mL
Peng F 2022 [84]	2020–2021	China	Prospective study	143 LC, 148 hepatitis B virus (HBV)-related hepatocellular carcinoma	291	NA	NA
Hadi H 2022 [85]	2021–2022	Malaysia	Cross-sectional study	HCC—in relationship with 26 HBV, 12 NASH, 2 HCV, 123 nonmalignant high-risk liver cirrhosis	163	36.7 mAU/mL	14.2 ng/mL

NA = non-available, LC = liver cirrhosis, CHB = chronic hepatitis B, AFP = alpha-fetoprotein, NASH = non-alcoholic steatohepatitis, NAFLD = nonalcoholic fatty liver disease, hepatitis B virus = HBV, hepatitis C virus = HCV, NBNC-HCC patients = non-B, non-C hepatocellular carcinoma patients, HCC = hepatocellular carcinoma.

**Table 2:** Global HCC—Accuracy of AFP, PIVKA II for HCC diagnostic.

Study	AUC PIVKA II	AUC AFP	Se PIVKA II %	Sp PIVKA II %	Se AFP %	Sp AFP %
Schotten C 2021 [53]	0.920	0.890	-	-	-	-
Choi J 2019 [54]	0.710	0.770	48.00	86.00	62.00	87.00
Best 2020 [55]	0.870	0.880	-	-	-	-
Wu M 2020 [56]	0.634	0.798	29.80	97.20	77.30	71.10
Malov SI 2021 [57]	0.760	0.630	54.60	88.60	45.50	94.50
Chan 2022 [58]	0.908	0.880	86.90	83.70	51.80	98.10
Chalasani 2021 [59]	-	0.840	-	-	46.00	88.00
Basile 2020 [12]	0.790	0.900	-	-	-	-
Nouso K 2019 [60]	-	0.885	-	-	81.80	82.60
Hemken P 2019 [61]	0.870	0.880	86.00	72.00	86.00	77.00
Unic A 2018 [62]	0.903	-	81.25	96.77	-	-
Liu Z 2020 [63]	0.731	-	50.60	94.30	-	-
Loglio A 2020 [65]	0.776	0.750	64.00	91.00	56.00	94.00
Caviglia 2020 [1]	0.790	0.737	68.00	84.00	72.00	66.00
Caviglia 2021 [21]	0.853	0.763	75.00	85.70	76.40	68.90
Chen 2018 [66]	0.820	0.720	65.20	90.00	43.70	90.00
Degasperi E 2021 [69]	0.780	0.630	76.00	79.00	29.00	97.00
Qi F 2020 [71]	0.835	0.810	83.50	71.60	73.60	80.70
Guan MC 2022 [73]	0.869	0.763	74.80	91.00	52.50	97.40
Si YQ 2020 [74]	0.901	0.765	81.20	88.50	51.50	89.70
Ji J 2021 [75]	0.932	-	84.08	90.43	61.33	91.15
Wang G 2020 [76]	0.925	0.745	86.80	90.20	52.10	91.40
Bhatti 2020 [6]	0.720	0.830	72.00	60.00	77.00	77.00
Wang Q 2019 [77]	0.913	0.744	81.30	93.60	64.80	77.20
Li T 2019 [78]	0.890	0.900	84.20	82.00	85.30	85.60
Feng H 2021 [79]	0.900	0.770	83.93	91.50	64.29	90.20
Nguyen HB 2022 [80]	0.925	0.910	91.00	76.00	73.00	92.00
Lee Q 2021 [81]	0.836	0.799	68.4	98.40	57.60	93.50
Chen 2020 [82]	0.935	0.826	85.00	93.00	84.10	70.90
Xu F 2021 [83]	0.900	0.790	89.00	91.70	68.80	87.60
Hadi H 2022 [85]	0.905	0.869	90.00	82.10	75.00	93.50

AFP = alpha-fetoprotein, AUC = area under ROC curve, NA = not available, DCP = PIVKA II = protein induced by vitamin K absence or antagonist-II, Se = sensitivity, Sp = specificity.

**Table 3:** Early HCC—Accuracy of AFP, PIVKA II for early HCC diagnosis.

Study	No	AUC PIVKA II	AUC AFP	Se PIVKA II %	Sp PIVKA II %	Se AFP %	Sp AFP %
Schotten C 2021 [53]	70	0.830	0.860	-	-	-	-
Choi J 2019 [54]	31	-	0.800	-	-	-	-
Wu M 2020 [56]	113	0.613	0.723	25.70	97.20	67.30	71.70
Chalasani 2021 [59]	81	-	0.780	-	-	-	-
Liu Z 2020 [63]	62	0.685	-	43.50	94.30	-	-
Song T 2021 [64]	88	0.747	0.794	55.70	88.40	65.90	88.40
Caviglia 2020 [1]	115	0.766	0.708	65.00	84.00	67.00	66.00
Caviglia 2021 [21]	47	0.810	0.704	-	-	-	-
Chen 2018 [66]	94	0.730	0.620	48.30	90.00	30.60	90.00
Piratvisuth T 2022 [67]	125	0.790	0.834	56.00	-	53.60	-
Song T 2020 [68]	100	0.750	0.760	60.00	84.70	51.50	92.50
Guan MC 2022 [73]	60	0.851	0.752	75.00	89.60	46.70	97.40
Wang G 2020 [76]	94	0.851	0.617	72.30	90.20	33.00	92.40
Wang Q 2019 [77]	74	0.835	0.621	68.90	89.70	47.30	29.95
Li T 2019 [78]	95	0.890	0.900	84.20	82.00	85.30	85.60
Peng F 2022 [84]	59	0.871	0.599	61.20	95.80	28.81	97.90

No = number of early HCC cases, AFP= alpha-fetoprotein, AUC = area under ROC curve, NA= not available, DCP = PIVKA II protein induced by vitamin K absence or antagonist-II, Se = sensitivity, Sp = specificity.

## Data Availability

Not applicable.
